# Tislelizumab plus nimotuzumab is effective against recurrent or metastatic oral squamous cell carcinoma among patients with a performance status score ≥ 2: a retrospective study

**DOI:** 10.3389/fonc.2023.1273798

**Published:** 2024-01-16

**Authors:** Wen-Jie Wu, Pu-Gen An, Yi-Wei Zhong, Xiao Hu, Lin Wang, Jie Zhang

**Affiliations:** ^1^ Department of Oral and Maxillofacial Surgery, Peking University School and Hospital of Stomatology, Beijing, China; ^2^ National Center of Stomatology & National Clinical Research Center for Oral Diseases, Beijing, China; ^3^ Central Laboratory, Peking University School and Hospital of Stomatology, Beijing, China

**Keywords:** tislelizumab, nimotuzumab, immunotherapy, oral squamous cell carcinoma, performance status score

## Abstract

**Objectives:**

The efficacy of treatments targeting recurrent or metastatic head and neck squamous cell carcinoma are unsatisfactory in practice for patients with a ECOG PS score ≥ 2. Thus, this study retrospectively evaluated the safety and efficacy of a programmed cell death 1 inhibitor (tislelizumab) combined with an epidermal growth factor receptor inhibitor (nimotuzumab) in treating patients with a PS score ≥ 2 who suffer from recurrent or metastatic oral squamous cell carcinoma (OSCC).

**Materials and methods:**

Fifteen patients were treated with tislelizumab (200 mg IV Q3W) and nimotuzumab (200 mg IV Q3W). Programmed cell death-ligand 1 (PD-L1) expression in tumor biopsies was assessed with immunohistochemistry. Whole-exome sequencing was used to evaluate treatment efficacy based on PD-L1 expression and gene mutation.

**Results:**

At a median follow-up of 9.6 months, median overall survival was 10.1 months, and median progression-free survival was 4.0 months. Overall response rate was 40%, with 6/15 patients achieving partial response. Eight patients exhibited nine adverse events, eight out of nine being grade 2 and the remaining being grade 3. Whole-exome sequencing showed that *DYNC1I2, THSD7A*, and *FAT1* mutations were associated with patient prognosis.

**Conclusion:**

Combination therapy involving tislelizumab plus nimotuzumab is a promising, low-toxicity treatment for recurrent or metastatic OSCC in patients with a PS score ≥ 2.

## Introduction

Programmed cell death 1 (PD-1) inhibitors are first- and second-line treatments for recurrent or metastatic head and neck squamous cell carcinoma (HNSCC) (1, 2). Several clinical trials have reported the overall efficacy of various PD-1 inhibitors. For example, objective response to first-line treatment of pembrolizumab + chemotherapy was 36%, whereas pembrolizumab alone yielded 17% objective response (2). Platinum chemotherapy with nivolumab resulted in a 13.3% response rate among patients (1). The epidermal growth factor receptor (EGFR) monoclonal antibody cetuximab similarly yielded a 13% response rate (3). Furthermore, cetuximab not only inhibited the target of EGFR, but also upregulated the expression level of PD-L1 in NK cells, enhancing the efficacy of immunotherapy. In addition, PD-L1 blockade could also enhance the antibody-dependent cellular cytotoxicity of cetuximab against HNSCC cells {Okuyama, 2023 #188492}. Two recent phase 2 clinical trials found that combination therapy with PD-1 inhibitors and EGFR inhibitors leads to higher response rates than monotherapy (4, 5). In a phase 2 trial, patients were treated with pembrolizumab plus cetuximab, resulting in 45% objective response rate (ORR), median overall survival (OS) of 18.4 months, and median progression-free survival (PFS) of 6.5 months (4). Another phase 2 trial showed that nivolumab plus cetuximab yielded 22% ORR in patients who had received prior therapy and 37% ORR in patients who had not. And the median OS was 11.4 months and 20.2 months, respectively (5).

Despite these promising results, HNSCC patients with an Eastern Cooperative Oncology Group Performance Status (ECOG PS) score ≥ 2 do not respond well in practice. Exacerbating the problem, patients with poor PS are excluded from large clinical trials.

Tislelizumab is an anti-PD-1 monoclonal immunoglobulin G4 antibody approved for the treatment of nine cancer types in multiple clinical trials (6). Nimotuzumab is a humanized anti-EGFR immunoglobulin G1 monoclonal antibody with mild toxicity (7). This study aimed to evaluate the safety and efficacy of tislelizumab plus nimotuzumab in patients with a ECOG PS score ≥ 2 who have recurrent or metastatic oral squamous cell carcinoma (OSCC).

## Materials and methods

### Study design

This retrospective study was performed in accordance with the Declaration of Helsinki. Procedures were approved by the Ethics Committee of Peking University School and Hospital of Stomatology and in compliance with international ethical standards (IRB number: PKUSSIRB-202059162). Written informed consent was obtained from all patients.

Patients were enrolled in this retrospective study from April 2021 to October 2022 according to the following inclusion criteria: (a) patients with recurrent or metastatic OSCC and (b) patients with a ECOG PS score ≥ 2. The exclusion criteria were as follows: (a) history of other tumors and (b) ineligibility for PD-1 inhibitors. Fifteen patients with recurrent or metastatic OSCC were enrolled and treated consecutively with tislelizumab plus nimotuzumab from September 2020 to February 2021. All participants received fixed-dose nimotuzumab (200 mg) and tislelizumab (200 mg) intravenously on the first day of each 3-week cycle until intolerable adverse events or progression disease (PD) or death occurred. For patient baseline data, see **Table 1**. **Supplementary Table 1** showed the treatment history of these 15 patients.

**Table 1:** Baseline characteristics.

		N=15 (%)
Age	Median	78 (38,85)
Gender	Male	4 (26.7%)
	Female	11 (73.3%)
ECOG	2	9 (60%)
	3	6 (40%)
Recurrence pattern	Local or regional recurrence only	14 (93.3%)
	Distant metastasis only	1 (6.7%)
PD-L1 CPS	<1	1 (6.7%)
	20>CPS≥1	10 (66.7%)
	≥20	4 (26.8%)
PD-L1 TPS	<1%	4 (26.8%)
	≥1%	11 (73.3%)
Treatment cycle	Median	7 (2-22)
Outcome	DCR PR	6 (40%)
	SD	6 (40%)
	PD	3 (20%)
State	Alive	3 (20%)
	Death	12 (80%)
Cause of death	Tumor progression	8 (53.3%)
	Pulmonary infection	2 (13.3%)
	Pulmonary infection + Hypokalemia	1 (6.7%)
	Intracranial infection	1 (6.7%)

ECOG, Eastern Cooperative Oncology Group; PR: Partial Response; SD: Stable Disease; PD: Progressive Disease; ORR: Objective Response Rate; DCR: Disease control rate; NA, not applicable;.

Samples for programmed cell death-ligand 1 (PD-L1) immunohistochemistry and whole-exome sequencing were obtained from biopsies of the primary tumor before treatment.

### Outcome definition and response assessment

Responses were assessed was based on Response Evaluation Criteria In Solid Tumors (RECIST1.1). Images were obtained every 8 weeks and evaluated by two experienced radiologists and an experienced surgeon. The ORR is the sum of the proportions of complete response (CR) and partial responses (PR). The PFS was defined as the period from the enrollment to the latest follow-up, PD, or death from any cause. The OS was defined as the period from the enrollment to the latest follow-up, or death from any cause during the follow-up. Adverse events (AE) were assessed according to Common Terminology Criteria for Adverse Events version 5 (CTCAE V5.0). The primary endpoint was OS. Secondary endpoints included ORR, PFS, and AE.

### PD-L1 immunohistochemistry assay

The 22C3 pharmDx assay (Agilent Technologies, Santa Clara, CA, USA) was used for PD-L1 staining. Immunohistochemistry was performed following manufacturer protocol. All specimens from patient tumors were fixed with formalin, then embedded in paraffin and sliced into 4 μm sections. Antigens were retrieved using a target retrieval solution (pH 6.1) at 97°C for 20 min and washed with a wash buffer. Slides were then incubated with specific primary antibodies (mouse anti-human PD-L1 monoclonal antibody, clone 22C3) and washed three times with a wash buffer. Next, they were incubated with secondary antibodies (rabbit anti-mouse immunoglobulin G polymer) at room temperature and washed three times with a wash buffer. Slides were stained with 3,3’-diaminobenzidine tetrahydrochloride to detect PD-L1 presence and counterstained with hematoxylin to visualize nuclei. The comprehensive positive score (CPS) was defined as the number of PD-L1-positive cells (tumor cells, lymphocytes, and macrophages) divided by the total number of tumor cells × 100. The tumor cell proportion score (TPS) was defined as the number of PD-L1-positive tumor cells divided by total number of tumor cells × 100%.

### Whole-exome sequencing

#### DNA extraction

Informed consent was obtained from patients for genetic analysis. Genomic DNA was extracted from tumor samples and paired peripheral blood samples using the Library Extraction Kit (MyGenostics, Beijing).

#### DNA library preparation

At least 3 µg of DNA was used to construct indexed Illumina libraries, following manufacturer protocol (MyGenostics). Fragments 350–450 bp in size, including adapter sequences, were selected for DNA libraries. Validation was performed with a Nanodrop 2000 spectrophotometer (Thermo Fisher, USA) and the Agilent 2100 Bioanalyzer (Agilent, USA).

### Targeted gene capture and sequencing

Total coding sequences of genes were selected via gene capture using the GenCap custom enrichment kit (MyGenostics). Paired-end reads (150 bp) were sequenced on a NextSeq 500 sequencer (Illumina, San Diego, CA, USA) for library construction.

### Data analysis

After sequencing, low-quality reads (quality score ≥ 20) were filtered out. Clean reads were aligned to the human reference genome (hg19) with the Burrows-Wheeler Aligner. Single nucleotide polymorphisms and insertions or deletions were identified using the Genome Analysis Toolkit, while Delly determined structural variations. Copy number variants were detected with the CNVkit, based on the depth distribution of reads compared with the reference genome.

### Statistical analysis

The Kaplan–Meier method was used for analyses of PFS and OS. Differences between groups were compared with the use of the stratified (unweighted) log-rank test. An 95% CI was estimated for PFS and OS. The *P* values are two-tailed and P < 0.05 was considered statistically significant. All analyses were performed in GraphPad Prism (version 9) and IBM SPSS (version 24). Heatmaps were created with the R package “Pheatmap” in R Studio.

## Results

### Efficacy evaluation

**Figure 1:**
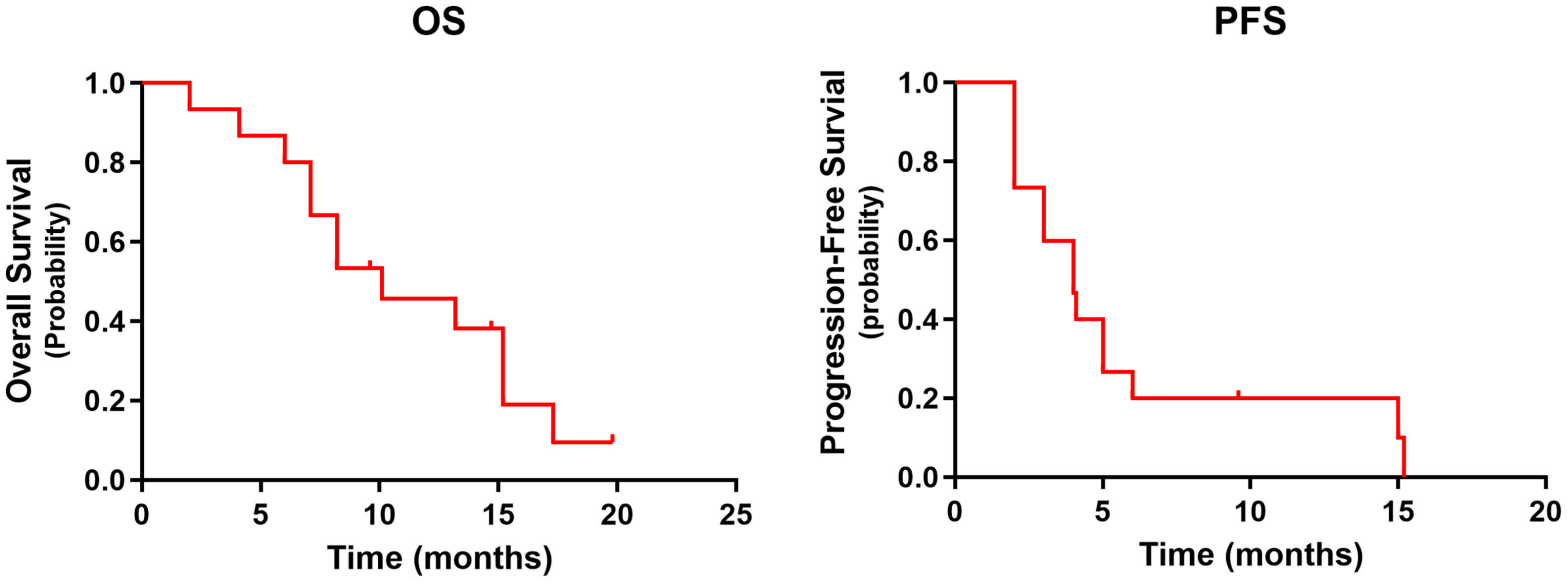
The OS and PFS in 15 patients. OS, overall survival; PFS, progression-free survival.

**Figure 2:**
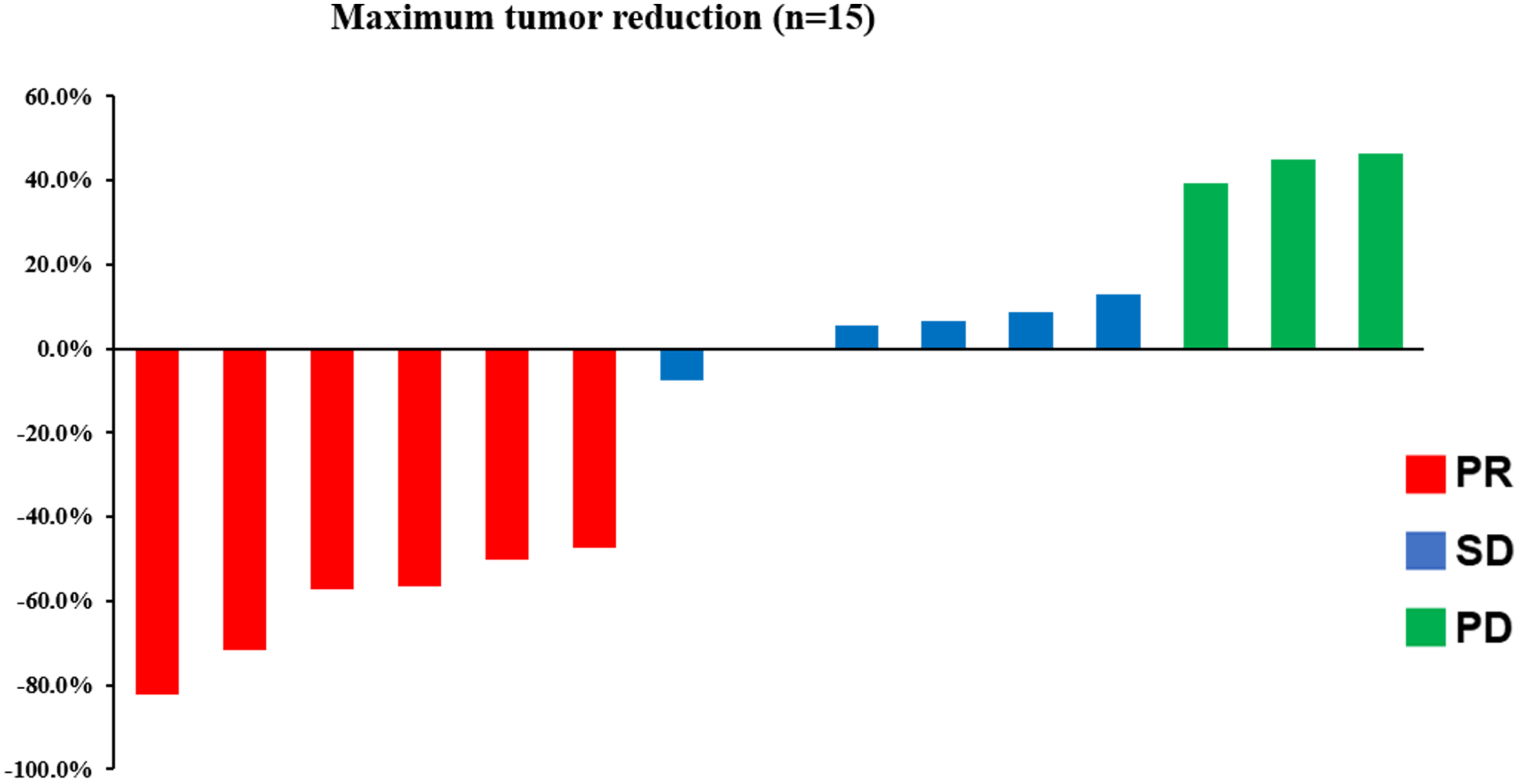
The therapy outcome of 15 patients.

**Figure 3:**
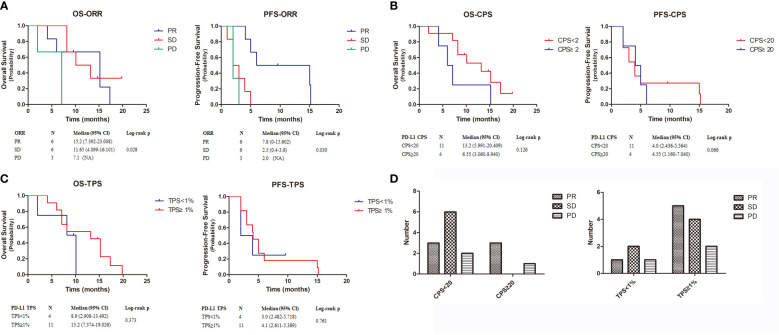
Kaplan-Meier estimates the OS and PFS of 15 patients. **(A–C)**. The OS and PFS of ORR, CPS and TPS. **(D)**. The relationship between CPS and TPS and patient treatment outcome. OS, overall survival; PFS, progression-free survival.

Median follow-up was 9.6 months (range: 2–15.2 months) at the data cutoff of November 30, 2022. Average patient age was 78 years, and most were women (**Table 1**). Median OS was 10.1 months (95% CI = 4.6–15.6 months), and median PFS was 4.0 months (95% CI = 2.0–6.0 months) (**Figure 1**). Among all participants, 12 patients (80%) responded to treatment (**Supplementary Figure 1**), six of them (40%) partially (PR). The best result was tumor shrinkage by 82.1% (**Figure 2**). Six patients (40%) had stable disease (SD), but three (20%) had progressive disease (PD). One patient had an SD status for 2 months and then received chemotherapy. The ORR was 40% and median OS was 15.2 months (95% CI = 7.4–23.0 months), 11.65 months (95% CI = 4.1–16.1 months), and 7.1 months (95% CI = not available [NA]) in the PR, SD, and PD groups, respectively, with significant differences between groups (p = 0.028). Median PFS also significantly different between groups (p = 0.030), being 7.8 months (95% CI = 0–15.602 months), 2.5 months (95% CI = 0.4–3.6 months), and 2.0 months (95% CI = NA), respectively (**Figure 3A**).

### Adverse events

The 15 patients generally responded well to treatment, with nine adverse events (AEs) in 8 patients (53.3%) (**Table 2**). Grade 2 acneiform rash had the highest incidence (26.7%), followed by hypothyroidism (13.3%), interstitial pneumonia (13.3%), and myocarditis (6.7%). Notably, one patient developed pneumonia after three cycles of combined treatment and was then treated with nimotuzumab alone for 16 cycles; her status was maintained at PR. One patient discontinued treatment after two cycles due to pneumonia and underwent chemotherapy. Tumor progression was observed in this patient. One patient discontinued combined treatment after 2 months due to myocarditis and was treated with cardiac support.

**Table 2:** Adverse Events.

	Grade 2	Grade 3
Rash acneiform	4 (26.7%)	
Hypothyroidism	2 (13.3%)	
Interstitial pneumonia	2 (13.3%)	
Myocarditis		1 (6.7%)

### Efficacy analyses based on PD-L1 immunohistochemistry

To further explore combined treatment efficacy, we evaluated OS and PFS based on CPS and TPS. Eleven individuals (73.2%) had PD-L1 CPS < 20, including one patient with PD-L1 CPS < 1. Four patients (26.8%) had PD-L1 CPS ≥ 20. Median OS was 13.2 months (95% CI = 5.991–20.409 months) and 6.55 months (95% CI = 3.060–8.940 months) in patients with PD-L1 CPS < 20 and PD-L1 CPS ≥ 20, respectively. Median PFS was 4 months (95% CI = 2.436–5.564 months) and 4.55 months (95% CI = 1.160–7.040 months) in the PD-L1 CPS < 20 and PD-L1 CPS ≥ 20 groups, respectively. There was no significant difference in OS (*p* = 0.126) and PFS (*p* = 0.066) (**Figure 3B**). Next, we examined patients based on TPS. Four patients had TPS < 1% and 11 had TPS ≥ 1%. Median OS was 8.9 months (95% CI = 2.908–13.492 months) and 13.2 months (95% CI = 7.37–419.026 months) in the PD-L1 TPS < 1% and PD-L1 TPS ≥ 1% groups, respectively. Median PFS in the two groups was 3.0 months (95% CI = 2.482–5.718 months) and 4.1 months (95% CI = 2.611–5.389 months). Neither OS (*p* = 0.373) nor PFS (*p* = 0.761) differed between groups (**Figure 3C**).

Additionally, we identified differences in treatment outcomes between the CPS/TPS groups (**Figure 3D**). The amount of patients with SD was significantly higher when CPS < 20 than when CPS ≥ 20. The amount of patients with PR was higher when TPS ≥ 1% than when TPS < 1%.

### Efficacy analyses based on mutations

**Figure 4:**
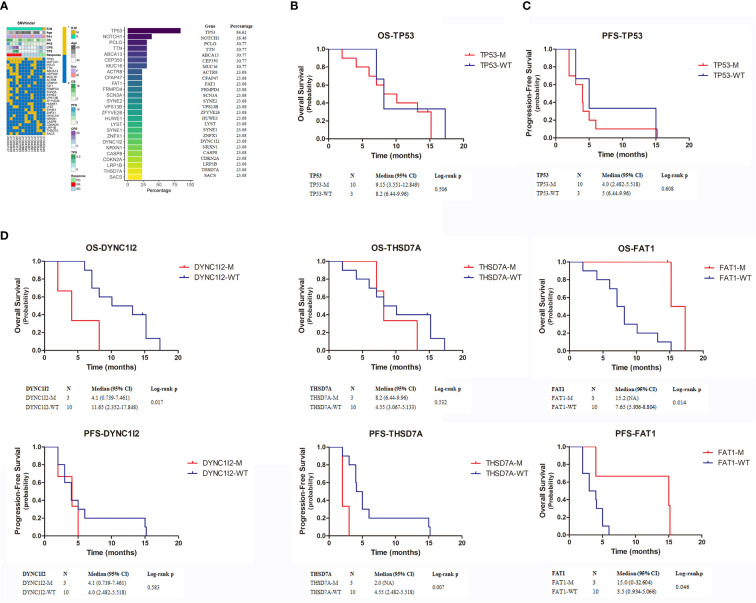
**(A)** The top 26 genes with the highest mutation rate in exon sequencing. **(B–D)** Kaplan-Meier estimates the OS and PFS of patients with TP53, DYNC1I2, THSD7A and FAT1 mutation. OS, overall survival; PFS, progression-free survival.

We performed whole-exome sequencing on tumor tissue and venous blood from 13 patients to clarify the influence of key genes on treatment efficacy (**Figure 4**). We found 26 mutations shared by more than three patients: 11 had *TP53* mutations; 5 had *NOTCH1* mutations; 4 had *PCLO, TTN, ABCA13, CEP350*, and *MUC16* mutations; and 3 had mutations in all of the following genes: *CFAP47, FAT1, FRMPD4, SCN3A, SYNE2, VPS13B, ZFYVE26, HUWE1, LYST, SYNE1, ZNFX1, DYNC112, NRXN1, CASP8, CDKN2A, LRP1B, THSD7A*, and *SACS* mutations. Next, we analyzed the prognosis of patients with mutations. Median OS and PFS in patients with mutated *TP53* were 9.15 months (95% CI = 3.551–12.849 months) and 4.0 months (95% CI = 2.482–5.518 months), respectively. Median OS and PFS of patients with wild-type *TP53* were 8.2 months (95% CI = 6.44–9.96 months) and 5 months (95% CI = 1.799–8.201 months), respectively. Neither OS nor PFS differed significantly between patients with mutated and wild-type *P53* (**Figures 4B, C**) (*p* = 0.506 and *p* = 0.608, respectively). In contrast, only 3genes, *DYNC1I2, THSD7A*, and *FAT1*, were associated with patient prognosis (**Figure 4D**). *DYNC112* was associated with median OS (*p* = 0.017), which was 4.1 months (95% CI = 0.739–7.461 months) and 11.65 months (95% CI = 2.352–17.848 months) in mutation and wild-type groups, respectively. *THSD7A* was associated with median PFS (*p* = 0.007), which was 2.0 months (95% CI = NA) and 4.55 months (95% CI = 2.482–5.518 months) in the mutation and wild-type groups, respectively. Finally, *FAT1* was associated with OS (*p* = 0.014) and PFS (*p* = 0.046). Median OS in patients with mutated and wild-type *FAT1* was 15.2 months (95% CI = NA) and 7.65 months (95% CI = 5.93–68.804 months), respectively. Median PFS was 15 months (95% CI = 0.32–32.604 months) and 3.5 months (95% CI = 0.934–5.066 months).

## Discussion

Combining chemotherapy with targeted therapy or immunotherapy can achieve an objective remission rate of >35% in recurrent or metastatic HNSCC (2, 8). However, treatment remains challenging for patients with a PS score ≥ 2 who cannot tolerate routine chemotherapy. In an observational study of nivolumab treatment in patients with recurrent or metastatic HNSCC, median OS was 9.2 months in individuals with a PS score of 0–1 and 4.0 months in those with a PS score > 2 (9). Similarly, the HANNA study showed that median OS was 25.6 months in patients with a PS score of 0 and 5.7 months in patients with a PS score > 2 (10). A phase 2 trial testing cetuximab plus weekly paclitaxel as first-line therapy for recurrent or metastatic HNSCC showed that median OS was 18.6 months in patients with a PS score of 0 and 7.3 months in patients with a PS score of 2 (11). These studies demonstrated that the PS score is an important prognostic factor, especially under anti-PD-1 monotherapy. In our study, PS score ≥ 2 patients had longer median OS and PFS than in other studies, indicating that tislelizumab plus nimotuzumab was relatively effective and safe. This drug combination may be a transitional treatment for PS score ≥ 2 patients who do not tolerate conventional chemotherapy. If combination treatment is effective, chemotherapy may be added to improve remission rates. However, causes of death in these patients are more likely to be systemic diseases than local ones, so the addition of a powerful treatment such as chemotherapy must be considered with caution.

Among chemotherapy-free options, a PD-1 inhibitor combined with an EGFR inhibitor has been very effective against recurrent or metastatic HNSCC. In a phase 2 trial for these cancers, patients were treated with pembrolizumab plus cetuximab, resulting in 45% ORR, median OS of 18.4 months, and median PFS of 6.5 months (4). Another phase 2 trial showed that nivolumab plus cetuximab yielded 22% ORR in patients who had received prior therapy and 37% ORR in patients who had not. Respectively, median OS was 11.4 months and 20.2 months (5). The 40% ORR in our study was consistent with these previous studies, although median OS and PFS were shorter. In the Keynote-048 trial, anti-PD-1 monotherapy was more effective in patients with metastases than in those with only recurrence, whereas EGFR inhibitors provided more clinical benefit for the latter (2). Combination therapy could be an alternative for patients with only recurrence.

Our study showed that tislelizumab plus nimotuzumab was relatively well-tolerated. Eight out of 15 patients experienced grade 2–3 AEs (mostly grade 2), with acneiform rash and hypothyroidism being the most common. However, in other clinical studies using cetuximab, AEs were often grade 3 or even grade 4. The most common AEs were rash, hypomagnesemia, and oral mucositis (2, 12, 13).

Nimotuzumab and cetuximab are both EGFR inhibitors. Although a higher dose of nimotuzumab is required to achieve effective outcomes, the drug has low toxicity. Nimotuzumab has low affinity for EGFR and only exhibits satisfactory activity in cells with higher EGFR expression, thus reducing its effect on healthy epithelial tissue cells (14). Nimotuzumab has performed well in clinical trials, improving patient survival and causing few adverse reactions (15, 16). Nimotuzumab combined with PD-1 inhibitors could be a treatment option that causes fewer AEs than PD-1 inhibitor monotherapy.

The Keynote-048 revealed that PD-L1 is a good biomarker for predicting ORR and OS in anti-PD-1 monotherapy for recurrent or metastatic HNSCC (2). In CheckMate-141 trials, among patients using nivolumab alone, patients with PD-L1 expression had a higher ORR than PD-L1 non-expressors (17 (CI, 10.7–26.8; N=96) vs 11.8 (CI, 5.6–21.3; n=76)). While, there seemed to be no significant difference between PD-L1 expressors and non-expressors in OS (17). In contrast, our study found that patients in the CPS < 20 group had a higher median OS than patients in the CPS ≥ 20 group (13.2 months vs. 6.55 months). Patients with CPS ≥ 20 also had slightly higher median PFS than patients with CPS < 20 (4.55 months vs. 4.00 months). Comparable results were reported from a phase 2 trial for recurrent or metastatic HNSCC (5). For patients treated with nivolumab plus cetuximab, median OS in the CPS < 20 group was 19.9 months, significantly higher than in the CPS ≥20 group (10.7 months) or the CPS < 1 group (8.9 months). Median PFS of Patients in the CPS ≥ 20 group also had higher median PFS than patients in the CPS < 20 group (5.6 months vs. 3.8 months) (5). Patients with higher CPS generally respond better to PD-L1 inhibitors, achieving longer OS and PFS (1, 2). However, this pattern was not borne out with combined treatment. Patients who test negative for PD-L1 may benefit more from a combination of PD-1 and EGFR inhibitors (5, 12), likely because EGFR pathway inhibition alters the immune structure of the tumor microenvironment (18, 19).

*TP53* has one of the highest mutation rates in HNSCC (20). *TP53* mutation has been associated with a decrease in immune cell infiltration and PD-L1 expression. Therefore, *TP53* mutation status may be a negative predictor of response to treatment with immune checkpoint inhibitors (21). In our study, *TP53* mutation status was not significantly associated with OS or PFS, suggesting that patients with these mutations may not benefit from immunotherapy. In contrast, *FAT1* mutation status was significantly associated with OS and PFS. Patients with *FAT1* mutations had higher median OS (*p* = 0.014) and PFS (*p* = 0.046). *FAT1* encodes tropocadherin, a protein that regulates intercellular adhesion and extracellular matrix structure. *FAT1* mutations are the most common in squamous cell carcinoma, especially OSCC (30–40%) (2013). In HNSCC, *FAT1* mutations induce EMT status, thereby promoting tumor occurrence, progression, invasiveness, and metastasis (22). In OSCC, therapy targeting *FAT1* successfully inhibited tumor progression and increased sensitivity to chemotherapy (23). In HNSCC cell lines, knocking out the *FAT1* gene could reduce the expression of pEGFR, pHER2, and pERK proteins, meaning to inactivate the EGFR signaling axis. In clinical research, there was a significant correlation between the expression of FAT1 and EGFR in SCC of the lung, cervix, and head and neck, with *FAT1* more commonly seen in HPV (-) HNSCC. In summary, mutations in *FAT1* may lead to resistance to EGFR targeted therapy (24–26). Considering these findings, future studies should aim to further clarify the effects of *FAT1* on immunotherapy and targeted therapy.

Although we provided evidence supporting the efficacy of tislelizumab plus nimotuzumab in treating recurrent and metastatic OSCC, our study had several limitations. First, the sample size of 15 patients is inadequate compared with other clinical studies. Second, only one patient exhibited metastasis, meaning we could not fully evaluate the effect of our proposed combination therapy on such patients.

Nevertheless, similar to studies that have evaluated the use of combination therapies with PD-1 and EGFR inhibitors in recurrent or metastatic HNSCC, the use of tislelizumab plus nimotuzumab demonstrated satisfactory response rates and OS in patients with a ECOG PS score ≥ 2 who have recurrent or metastatic OSCC. The drug combination also exhibited low toxicity and was relatively safe.

## Conclusions

Our results suggest that tislelizumab in combination with nimotuzumab is a promising, low-toxicity therapy for recurrent or metastatic OSCC among patients with a ECOG PS score ≥ 2.

## Data availability statement

The original contributions presented in the study are publicly available. This data can be found here: https://dataview.ncbi.nlm.nih.gov/object/PRJNA907775?reviewer=vcmj5u5t8ijbnp3o784nh8h9r4.

## Ethics statement

The studies involving humans were approved by Ethics Committee of Peking University School and Hospital of Stomatology (IRB number: PKUSSIRB-202059162). The studies were conducted in accordance with the local legislation and institutional requirements. The participants provided their written informed consent to participate in this study.

## Author contributions

W-JW: Formal analysis, Software, Writing – original draft, Data curation, Validation. P-GA: Methodology, Formal analysis, Software, Writing – original draft. Y-WZ: Data curation, Writing – original draft, Investigation. XH: Investigation, Writing – original draft. LW: Formal analysis, Writing – original draft. JZ: Conceptualization, Funding acquisition, Methodology, Project administration, Writing – review & editing.

## Supplementary material

Supplementary Figure 1 |A 72-year-old male presented with bilateral cervical lymph node metastasis after surgery and radiotherapy for the cancer of the floor of the mouth. **(A)**. Bilateral cervical metastatic lymph nodes (the white arrow). **(B)**. The image PR was achieved after 3 cycles of tislelizumab plus nimotuzumab. **(C)**. H&E (10x). **(D)**. PD-L1 staining (the CPS was 2 and TPS was 2%, 10x) was performed using the 22C3 pharmDx assay (Agilent Technologies, Santa Clara, CA, USA).
