# Supplementary figures and images for: Tislelizumab plus nimotuzumab is effective against recurrent or metastatic oral squamous cell carcinoma among patients with a performance status score ≥ 2: a retrospective study
Source: Front Oncol. 2024 Jan 16;13:1273798. doi: 10.3389/fonc.2023.1273798 (PMC10824828; doi:10.3389/fonc.2023.1273798)

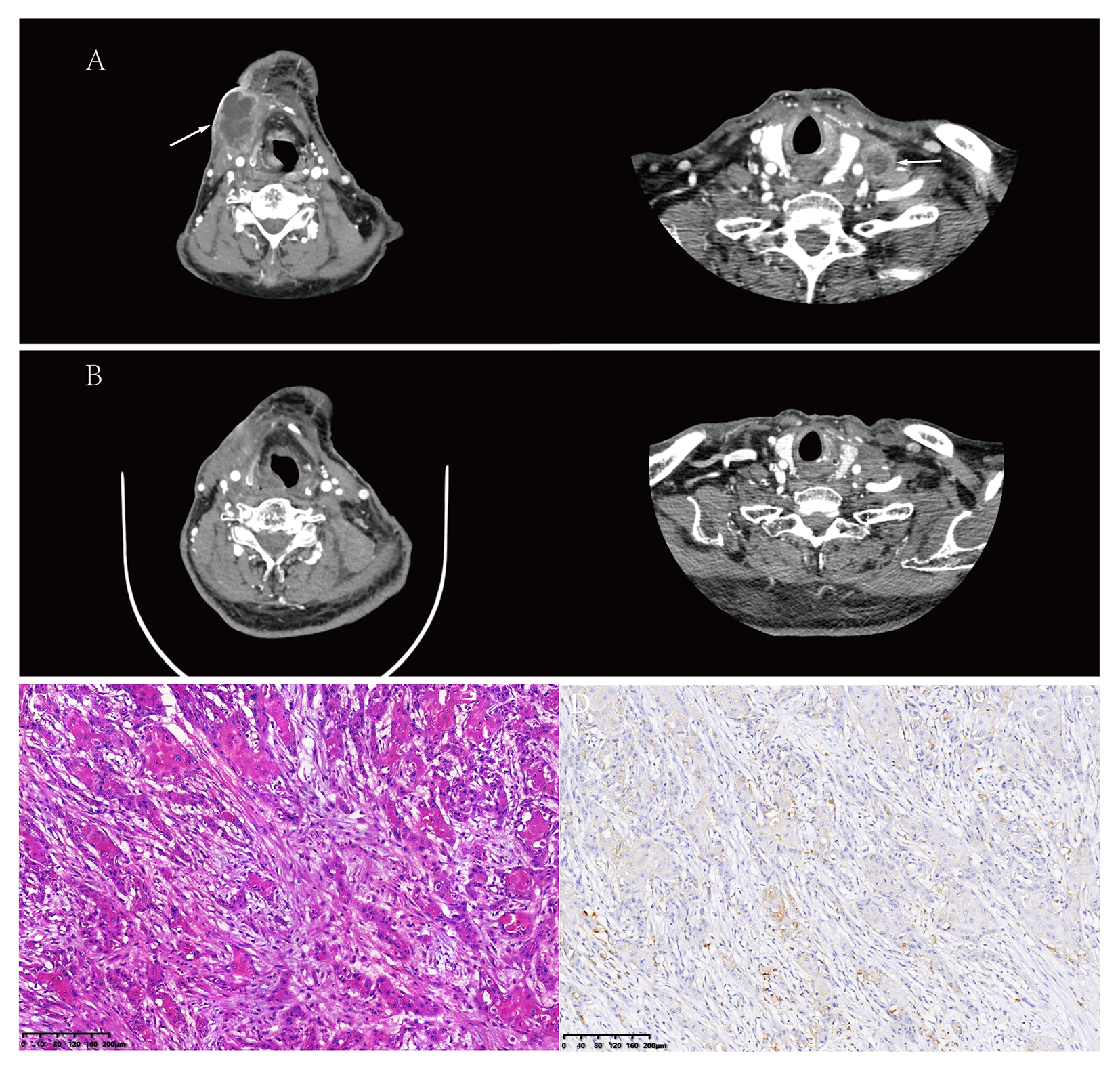

Supplement: Supplementary Figure 1 — |A 72-year-old male presented with bilateral cervical lymph node metastasis after surgery and radiotherapy for the cancer of the floor of the mouth. (A). Bilateral cervical metastatic lymph nodes (the white arrow). (B). The image PR was achieved after 3 cycles of tislelizumab plus nimotuzumab. (C). H&E (10x). (D). PD-L1 staining (the CPS was 2 and TPS was 2%, 10x) was performed using the 22C3 pharmDx assay (Agilent Technologies, Santa Clara, CA, USA). [file Image_1.tif]
