# Supplementary material for: Tislelizumab plus nimotuzumab is effective against recurrent or metastatic oral squamous cell carcinoma among patients with a performance status score ≥ 2: a retrospective study
Source: Front Oncol. 2024 Jan 16;13:1273798. doi: 10.3389/fonc.2023.1273798 (PMC10824828; doi:10.3389/fonc.2023.1273798)
Supplement: Supplementary file 2 [file Table_1.docx]

Table S1. The treatment history of these 15 patients.

| Patient number | Sites | Initial treatment | Recurrence/ Metastasis and Treatment  DFT (months) |
| --- | --- | --- | --- |
| 1 | Buccal mucosa | Surgery only | LR→Surgery→LR→T+N  28 4 |
| 2 | Mandibular gingiva | Surgery only | LR→T+N  6 |
| 3 | Floor of mouth | Surgery+RT | LR→T+N  6 |
| 4 | Maxilla gingiva and buccal mucosa | Surgery only | LR→Surgery+RT→LR→Surgery→LR→Surgery→  12 6 12  M(lung)→T+N  1 |
| 5 | Buccal mucosa | Surgery+RT | LR→T+N  30 |
| 6 | Oral tongue | Surgery only | LR (cervical lymph node)→Surgery+RT→LR→T+N  11 2 |
| 7 | Oral tongue | Surgery+RT | LR→T+N  7 |
| 8 | Buccal mucosa and mandibular gingiva, oral tongue; floor of mouth | Surgery only | LR→T+N  17 |
| 9 | Maxillary gingiva | RT | Surgery→LR→T+N  2 |
| 10 | Mandibular gingiva | Surgery only | LR→T+N  4 |
| 11 | Buccal mucosa and mandibular gingiva | Surgery only | LR→T+N  42 |
| 12 | Maxillary gingiva | RT | Surgery→LR→T+N  3 |
| 13 | Oral tongue | Surgery+RT | LR→T+N  38 |
| 14 | Buccal mucosa | Surgery only | LR→T+N  3 |
| 15 | Oral tongue | Surgery+RT | LR (cervical lymph node)→T+N  13 |

LR, local-regional recurrence; M, metastasis; T, tislelizumab; N, nimotuzumab; RT, radiotherapy; DFT: diseases-free time
